# 

**Published:** 1920-10-16

**Authors:** 


### THE HOSPITAL

The Modern Newspaper of Administrative Medicine and Institutional Life.

Telegraphic Address: OSPEDALE, RAND, LONDON. Telephone Number: 2734 GERRARD.

No. 1793, Vol. LXIX. | Saturday, October 16th, 1920. | PRICE TWOPENCE.

